# A Multi-level Memristor Based on Al-Doped HfO_2_ Thin Film

**DOI:** 10.1186/s11671-019-3015-x

**Published:** 2019-05-28

**Authors:** Lei Wu, Hongxia Liu, Jiabin Li, Shulong Wang, Xing Wang

**Affiliations:** Key Laboratory for Wide-Band Gap Semiconductor Materials and Devices of Education, School of Microelectronics, Xidian University, Xi’an, 710071 China

**Keywords:** Memristor, HfO_2_, Al doped, Multi-level memory

## Abstract

Non-volatile memory (NVM) will play a very important role in the next-generation digital technologies, including the Internet of things. The metal-oxide memristors, especially based on HfO_2_, have been favored by lots of researchers because of its simple structure, high integration, fast operation speed, low power consumption, and high compatibility with advanced (complementary metal oxide silicon) CMOS technologies. In this paper, a 20-level stable resistance states Al-doped HfO_2_-based memristor is presented. Its cycles endurance, data retention time, and resistance ratio are larger than 10^3^, > 10^4^ s, and > 10, respectively.

## Background

Although negative resistance phenomenon firstly was discovered by Hickmott in an Al/Al_2_O_3_/Au structure in 1962 [1], and Chua proposed the concept of memristor in 1971 [2]. It was not until Strukov et al prepared the TiO_2_-based memristor in 2008 [3] that people began to pay attention to the study on memristors. At present, researchers have prepared memristors with more than dozens of active resistive materials, including multiple complex oxides [4, 5], metal oxides such as ZnO [6], TiO_x_ [7], TaO_x_ [8], and two-dimensional materials [9, 10]. HfO_2_ has been used as high-k gate dielectrics in CMOS devices since its high reliability, fast operation speed, and low-power consumption [11, 12]. It is also preferred by researchers as a memristive material [13–15].

Multi-level memristor can be widely used as data storage [16–18], logical calculation [19], electronic synaptic device [20–23], and so on. Wang Y. [16] and Gao B. et al. [24] prepared multi-level memristors by doping HfO_2_ with Cu and Gd, respectively, but they can only create 4-level storage state, which is difficult to meet the demands of the applications. Therefore, the study on HfO_2_ multi-level memristors is of great significance.

## Methods

**Fig. 1:**
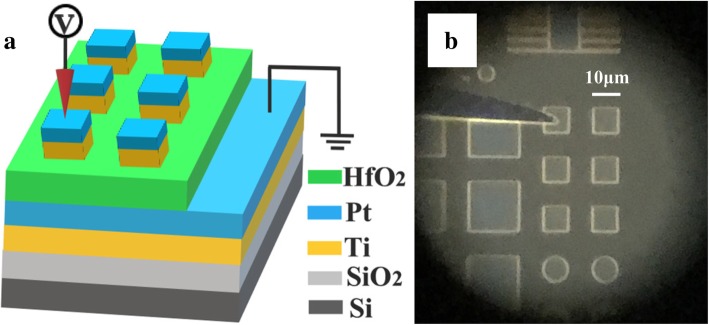
The structure of the devices. **a** 3D model of the memristors. **b** Optical microscopy of the devices

Ti/Al:HfO_2_/Pt device was fabricated as shown in Fig. 1a. The active cell area was defined by the square-shaped Ti top electrode (TE). A 20-nm Ti adhesive layer was deposited by direct current (DC) sputtering on a silicon substrate, then a 100-nm Pt film was deposited as a bottom electrode (BE). The 20-nm Al-doped HfO_2_ functional layer was deposited by the atomic layer deposition (ALD) reactor (R-150, Picosun, Espoo, Finland) at 300 °C with MeCp_2_HfMe(OMe) (denoted as HfD-04) as Hf precursor, and H_2_O as oxygen source [25]. The precursors were carried by high-purity N_2_ (> 99.999%) into the reactor chamber. Al-doped films were obtained by depositing one cycle of Al_2_O_3_ at every 8 cycles of HfO_2_ with the trimethylaluminum (TMA) as the Al source and H_2_O as oxygen source. The Al atomic concentration of 6.2% is detected by X-ray photoelectron spectroscopy (XPS, Axis Ultra DLD, Kratos Analytical, Manchester, UK) on a Theta 300 XPS system from Thermo Fisher. A 50-nm Ti film as TE and 100 nm Pt as covering layer were deposited by DC sputtering. Devices are obtained by patterning the TE by optical lithography and lift-off process. Figure 1b is the optical micrograph of the devices. We have prepared devices with different areas ranging from 5 μm × 5 μm to 500 μm × 500 μm.

## Results and Discussion

**Fig. 2:**
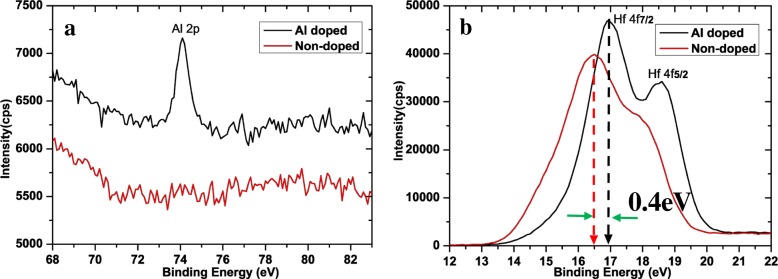
The XPS of Al-doped and non-doped devices. **a** Al 2p and **b** Hf 4f are compared

Figure 2 shows the XPS of Al-doped and non-doped devices. Comparing to the spectrograms of non-doped devices, Al-doped devices show a distinct 74.1 eV peak of Al 2p in Fig. 2a, and the binding energy of Hf 4f has a certain increase in Fig. 2b. The ratio of Hf 4f_5/2_ to Hf 4f_7/2_ also increased for the doped devices. It is consistent with the other reports [14, 26, 27]. Al atoms bond to HfO2 structure to form Hf-Al-O, which results in the weaker and more easily broken Hf-O bonds.

**Fig. 3:**
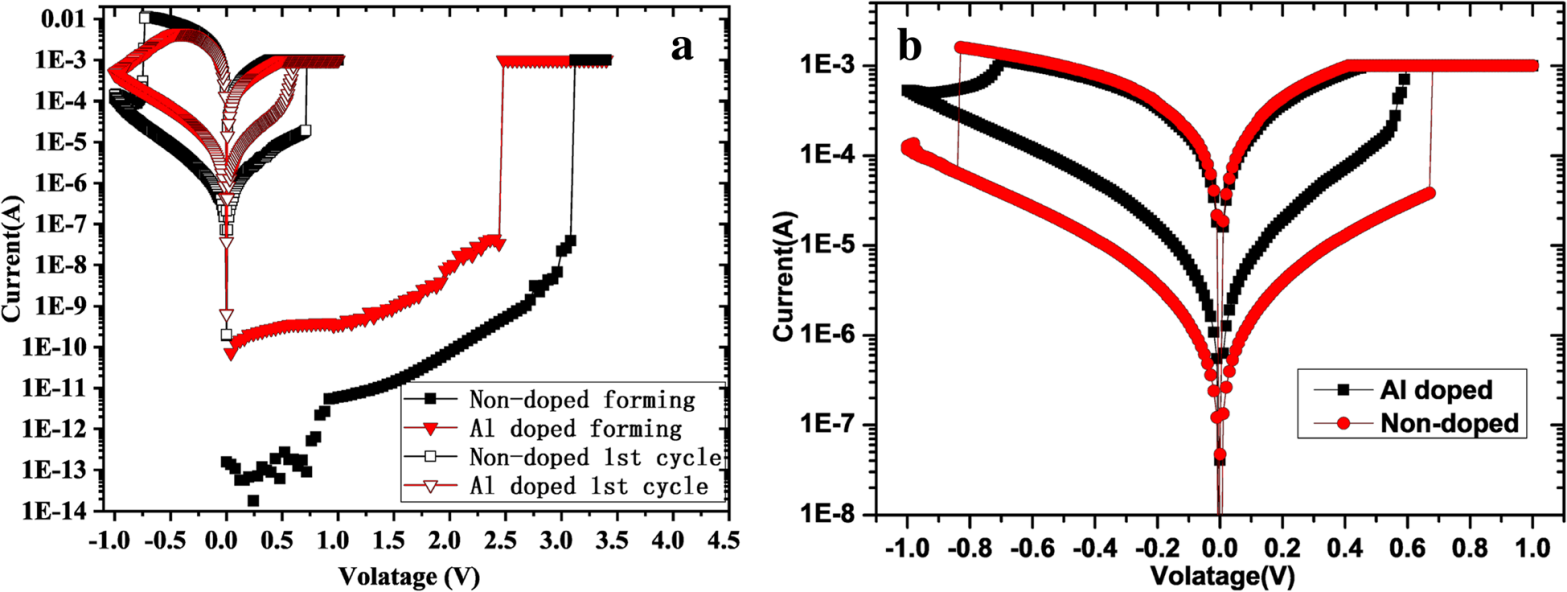
The I/V characteristics of the devices. **a** The forming process and first cycle. **b** The typical set/reset process

For all the electrical measurements, the Ti TE was biased while the Pt BE was grounded. DC sweeps were performed by using a B1500A parameter analyzer (Santa Clara, CA, USA) with a source/measurement unit, and pulse electrical measurements with a waveform generator/fast measurement unit are also used. All the devices show high-resistance state (HRS) before a necessary electric forming process. Figure 3a shows the forming characteristic of the 10 μm × 10 μm Al-doped and non-doped devices. A current compliance during forming is necessary to protect the devices from being damaged. The initial resistance and forming voltage of non-doped device is larger. The oscillation in the low-voltage region of the non-doped device is because the current is lower than the measuring limit of the instrument. The reset process after the forming step is motivated by applying a negative voltage, as shown in Fig. 3a, and then the first set process is motivated. As the voltage amplitude of reset increases, the current of both two devices increase to a maximum larger than the limited current of forming and then decrease. The HRS currents of both two devices are several orders larger than that of the initial state at the same voltage. It suggests that there is still conductive filament that cannot be fused completely after reset. The typical set/reset I–V curves in Fig. 3b shows both typology of these two devices is bipolar operation mode [28]. The switching ratio and the set/reset voltage of Al-doped device are smaller than those of non-doped device, but its resistance state transformation process is more gentle, suitable as a multi-value storage device.

**Fig. 4:**
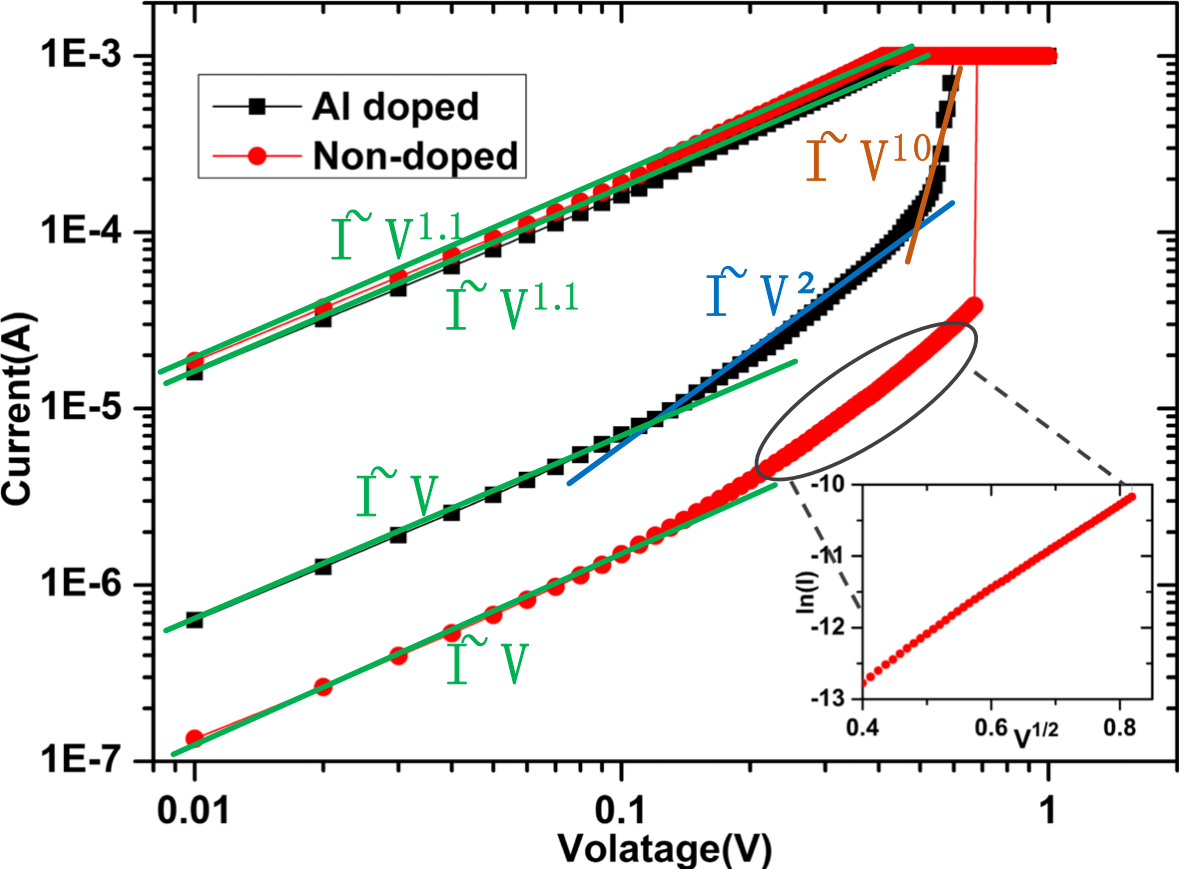
The curve fitting of SET process in the double logarithmic coordinates

To clarify the switching mechanisms of the devices, the I–V curves are replotted in double logarithmic scale in Fig. 4. For both kinds of devices, the low-resistance curve exhibits a linear Ohmic behavior, which indicates the formation of conducting filaments in HfO_2_ films during their setting [29, 30]. However, the high-resistance curves are quite different between these two kinds of devices. For the doped device, it is composed of three regions: the Ohmic region (I∝V), the Child’s law region (I∝V^2^), and the steep current increase region, which is accorded with the typical I–V characteristic of trap-controlled space charge limited current (SCLC) [31, 32]. The high-resistance curve of the non-doped device is composed of two regions: the Ohmic conduction (I∝V) at the low-voltage region, and the linear fit of the lnI-V^1/2^ at high-voltage region (the inset of Fig. 4), confirming the Schottky emission mechanism [15, 33].

According to the features above, the microscopic mechanisms of the memristors are summarized as follows. For undoped devices, as the positive voltage applied to the titanium electrode increases, more and more oxygen ions generate in the HfO_2_ and move toward the titanium electrode [34], producing titanium oxide [35]. At the same time, the oxygen vacancies accumulate at the interface between the platinum electrode and the HfO_2_, forming conductive filaments gradually [36]. Therefore, current increases gradually with the voltage. The devices turn into low-resistance state (LRS) when the oxygen vacancies conducting filaments connect the TE and BE. While the titanium electrode applied with a negative voltage, the oxygen ions combine with the oxygen vacancies at the HfO_2_/Pt interface [37], which leads to the lower oxygen vacancy concentration and the higher Schottky barrier. When the reset voltage reached, the conductive filament is broken and the device is changed to HRS.

For Al-doped devices, Al atoms bond to HfO2 structure to form Hf-Al-O result in the weaker and more easily broken Hf–O bonds. The formation energy of oxygen vacancy is reduced. Therefore, the doping devices have a smaller resistance and a lower transition voltage. In undoped films, oxygen vacancies tend to accumulate along the grain boundaries [38, 39]. As a result, conductive filaments are few and thick. The resistance of the devices varies greatly with the conduction and breakage of the conductive filaments. In the doped films, oxygen vacancies are easily formed near the impurity atoms [35, 40, 41]. The uniform distribution of a large amount of impurities in the thin film makes the conductive filaments be formed by oxygen vacancies more controllable. Therefore, it is easier to achieve multiple resistance values.

**Fig. 5:**
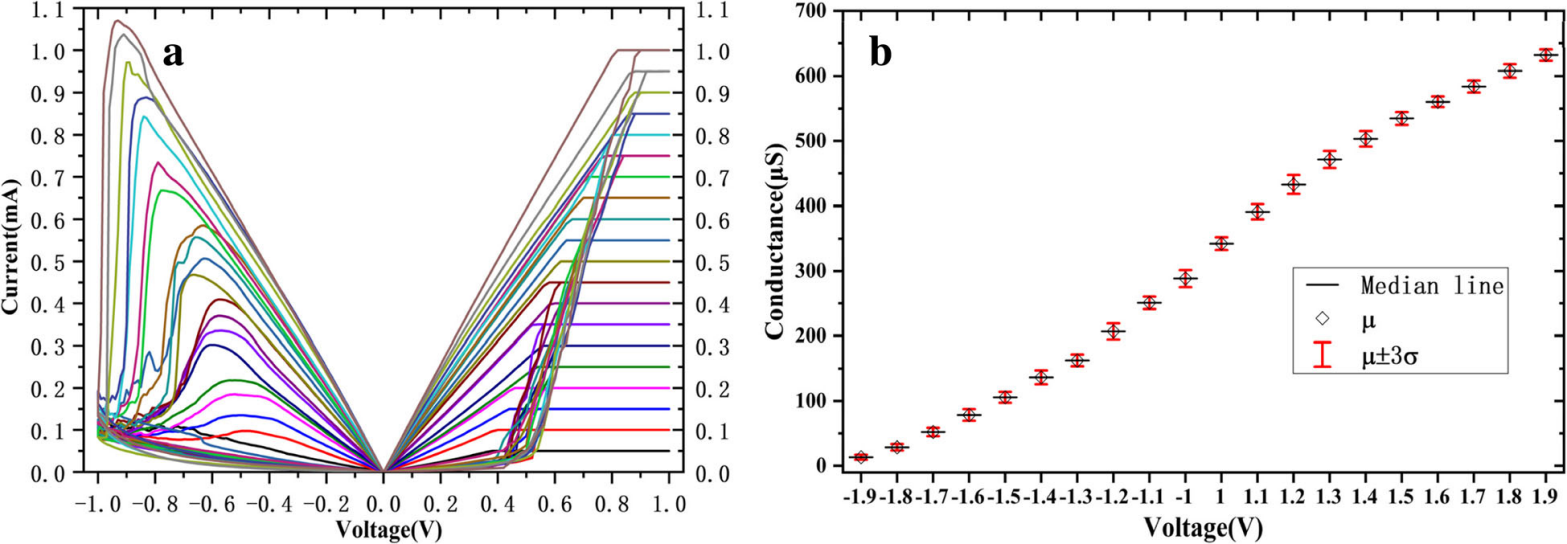
The multi-value storage of the devices. **a** Transform resistance states by setting compliance currents. **b** 20 stable resistance states obtained by setting pulse voltage amplitude The pulse width and interval are both 500 μs

The devices can be set to different steady resistance states by changing the current compliance of set process. Twenty stable resistance states are obtained by setting current compliance forming 0.5 mA to 10 mA with a step of 0.5 mA in Fig. 5a. As the resistance states set by DC sweep, the energy consumption is large, and the operation is complicated. On the other hand, the resistance values are easily locked in LRS when a large current compliance is used. This method is also unable to adjust the HRS. Twenty-level resistance states achieved by changing voltage amplitude of set and reset pulse. To avoid the possibility of current overshooting and set/reset failure, the voltage amplitude is limited between 1 V~1.9 V for SET and − 1 V~− 1.9 V for reset. It can be seen from the box diagram (Fig. 5b) that the allowed voltage range is divided into 20 values and the yield of the device is far exceeding the 3 σ level (99.73%). This is a common requirement in production. The disadvantage of this method is that the devices cannot be set directly from one HRS (LRS) to another HRS (LRS), but need to reset (set) to LRS (HRS) firstly, and then set to the target HRS (LRS). This increases the complexity and power consumption of the operation.

**Fig. 6:**
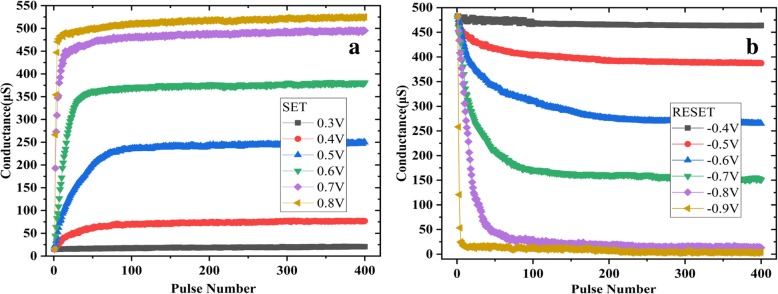
The resistance adjusted by consecutive pulses. **a** Set process and **b** reset process

**Fig. 7:**
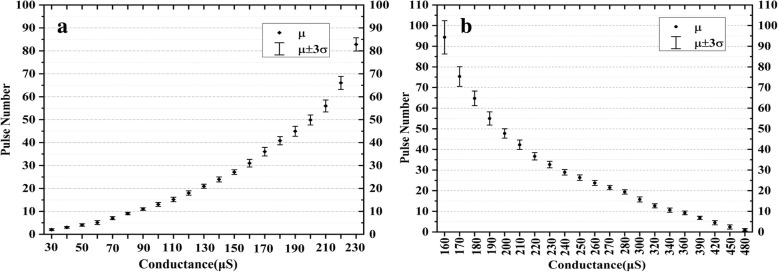
Pulse number needed to set (**a**) or reset (**b**) the devices to 20 different levels

A better approach is shown in Fig. 6. The device conductance is incrementally increased or decreased by consecutive pulses. The pulse duration and interval are both 10 μs. The conductance is measured by a 0.1 V read pulse after each set/reset pulse. As seen in Fig. 6, the number of pulses needed in order to set/reset the devices to different levels depends on the voltage applied. The different resistant statues with 20 levels are obtained through set and reset by selecting 0.5 V as SET voltage and − 0.7 V as reset voltage respectively (Fig. 7). The device is reset to a HRS by 10 − 0.9V consecutive pulses every time before setting to the target status or set to a LRS by 10 0.8-V consecutive pulses before adjustment. Considering the same status present at both the set and reset process, there are 35 different statuses obtained totally. The deviation of pulse number needed for the two adjacent resistance states of set (reset) exceeds the 3 σ level. The disadvantage is that if the resistance values of the devices change greatly, the pulse number needed will be large and the operation speed will be slow.

**Fig. 8:**
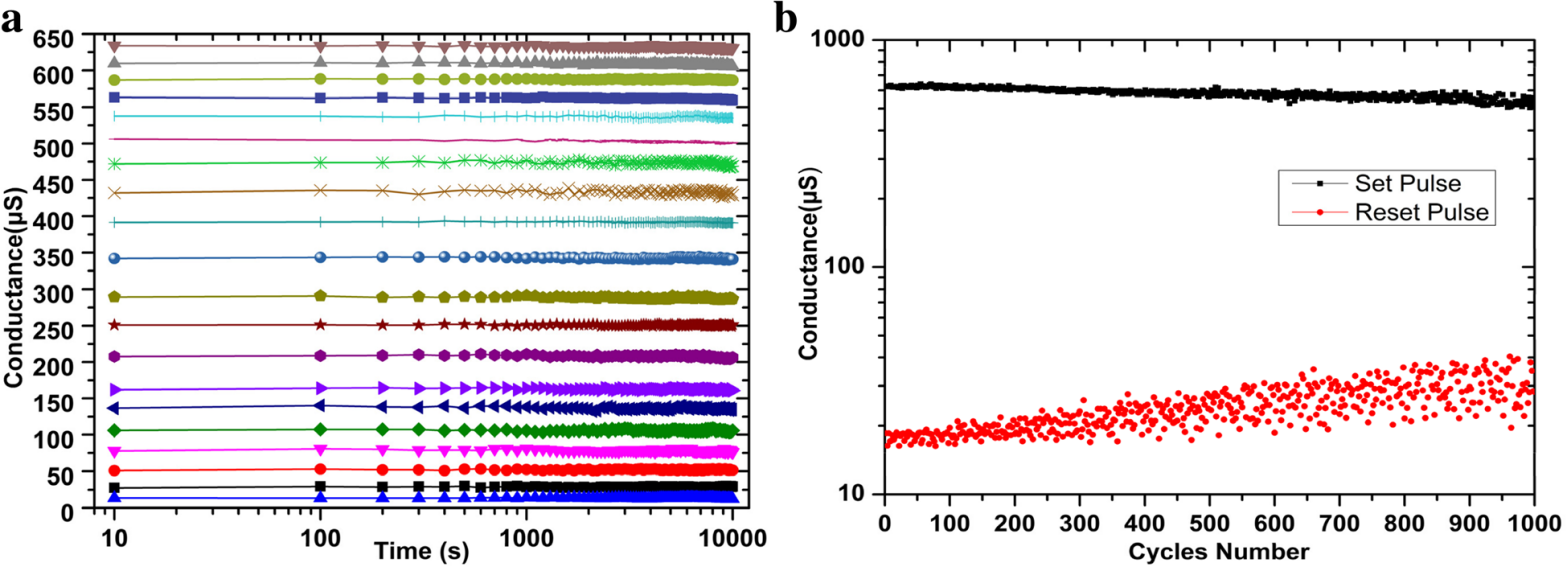
The reliability tests of the devices. **a** Data retention characteristics after set/reset pulse operations. **b** Cycles endurance curves for set/reset pulse operations

To test the data retention of the devices, 20 devices are set/reset to a series of different resistance values, and keeping them on a heating table at 85 °C [42]. The resistance values were measured with a voltage of 0.1 V every 100 s. It can be seen from Fig. 8a that the resistance of the devices maintains stable after 10^4^ s. In order to test the cycle reliability of the device, we repeated set and reset operation with a 1.8 V/500 μs set pulse and a − 1.8 V/500 μs reset pulse. After 10^3^ cycles, the switching ratio of the device is still greater than 10 in Fig. 8b.

## Conclusions

The proposed Al-doped HfO_2_ memristor shows a gradual and stable set/reset performance. By fitting the curve of set process of Al-doped and undoped devices, it is found that, in HRS, the undoped device follow Schottky emission mechanism, while the Al-doped device follow SCLC conductive mechanism. The microscopic physical mechanism of resistance change is also discussed. In addition, the multi-value storage of the device was confirmed by changing the compliance current, adjusting the set/reset pulse voltage amplitude and using the consecutive short pulses. Finally, we tested the reliability of the devices to prove that it has a data retention of more than 10^4^ s (85 °C) and a switching ratio greater than 10 after 10^3^ cycles.

## Data Availability

All data generated or analyzed during this study are included in this published article.

## Abbreviations

ALD: Atomic layer deposition
BE: Bottom electrode
CMOS: Complementary metal oxide silicon
HfD-04: MeCp_2_HfMe(OMe)Hf
HRS: High-resistance state
LRS: Low-resistance state
NVM: Non-volatile memory
SCLC: Space-charge-limited current
TE: Top electrode
TMA: Trimethylaluminum
XPS: X-ray photoelectron spectroscopy
